# Effect of Cs^+^ Doping on the Carrier Dynamics of MAPbI_3_ Perovskite

**DOI:** 10.3390/ma16176064

**Published:** 2023-09-04

**Authors:** Menghan Duan, Yunpeng Wang, Pingli Zhang, Luchao Du

**Affiliations:** 1 Institute of Atomic and Molecular Physics, Jilin University, Changchun 130012, China; 2 State Key Laboratory of Luminescence and Applications, Changchun Institute of Optics, Fine Mechanics and Physics, Chinese Academy of Sciences, Changchun 130033, China

**Keywords:** Cs^+^ doping MAPbI_3_ perovskite, transient absorption spectra, crystal structure, carriers dynamics

## Abstract

Organic inorganic perovskite materials have received increasing attention in the optoelectronic field because of their unique properties. The ultrafast dynamics of photogenerated carriers determine photoelectric conversion efficiency, thus, it is feasible to influence the dynamics behavior of photogenerated carriers by regulating A-site cations. This paper mainly used transient absorption spectra (TAS) technology to study the photogenerated carriers relaxation processes of organic–inorganic perovskite Cs_x_MA_1−x_PbI_3_ materials at different x values. Three sets of time constants were obtained by global fitting at different values of x. The experimental results showed that the crystal structure of perovskite could be affected by adjusting the Cs^+^ doping amount, thereby regulating the carrier dynamics. The appropriate amount of A-cation doping not only maintained the organic–inorganic perovskite crystal phase, but also prolonged the photogenerated carrier’s lifetime. The 10% Cs^+^ doping Cs_x_MA_1−x_PbI_3_ perovskite has potential for solar cell applications. We hope that our research can provide dynamics support for the development of organic–inorganic perovskite in solar cells.

## 1. Introduction

Organic–inorganic perovskite materials (ABX_3_) is a unique material; A^+^ represents organic cations (usually MA^+^ = CH_3_NH_3_^+^; FA = CH_3_(NH_2_)_2_^+^, etc.; B^2+^ = Pb^2+^, Sn^2+^, Ge^2+^, etc.; while X^−^ represents isohalogen ions = Cl^−^, Br^−^, I^−^, etc.). The inorganic structure and organic cations are connected by certain interaction forces; the inorganic structure is an octahedral structure [BX_6_]^4−^ formed by strong coordination bonds. In 2009, Kojima et al. reported the first liquid dye-sensitized solar cells (DSSCs) prepared by organic–inorganic hybrid perovskite (CH_3_NH_3_PbI_3_ or CH_3_NH_3_PbBr_3_); the DSSCs’ photoelectric conversion efficiency reached 3.8% [1]. In recent years, the perovskite photoelectric conversion efficiency has rapidly improved. Recently, the King Abdullah University of Science and Technology, Saudi Arabia, have produced a perovskite and silicon tandem solar cell with a photoelectric conversion efficiency of more than 33% [2]. Organic–inorganic plumbum halide perovskite has become one of the key photovoltaic materials and profits from its unique performance properties which attract attention, such as the large absorption coefficient of perovskite and the long carrier diffusion length; thus, it is mainly used in solar cell devices, light-emitting diodes, laser technology, and other research fields [3,4,5,6,7,8,9,10]. Organic–inorganic perovskite is unstable and usually degraded in light, humidity, and heat. The main cause of perovskite decomposition is the high volatility of organic CH_3_NH_3_^+^ cations [11]. To improve the structural stability, organic–inorganic perovskite, formed by mixing cations, has attracted scientists’ attention [12,13,14,15].

In order to improve the power conversion efficiency and the long-term stability of the material, the composition regulation of the CH_3_NH_3_PbI_3_ material is an effective strategy [16,17]. Recently, the substitution of organic cations in organic–inorganic perovskite materials has aroused much interest [18]. The choice of the A-site cation has important effects on the regulation of perovskite photoelectric properties. The perovskite performance of mixed A-cationic perovskite will improve relative to single-cationic perovskite [19]. In most perovskite, the electronic state of the perovskite [BX_6_]^4−^ octahedral structure determines the valence band maximum and conduction band minimum, and has an influence on the perovskite optical absorption and carrier transport performance, while the A-site cations are used to stabilize the perovskite octahedral structure [20]. Taya et al. demonstrated that doping Cs^+^ in MAPbI_3_ will result in an octahedral tilt increase and a band gap increase. The organic–inorganic perovskite, obtained by doping 12.5% of Cs^+^, is a potential candidate material for solar cells [21]. Imran et al. demonstrated that Cs^+^ can promote the organic–inorganic perovskite phase transition [22]. The perovskite crystal phase transition results in a series of physical properties changes, such as thermal, mechanical, electrical, optical, and other physical characteristics. The appropriate amount of Cs^+^ can stabilize the perovskite structure and improve the perovskite photoelectric conversion efficiency [23,24].

To develop the application of organic–inorganic perovskite materials in the photovoltaic field, such as solar cells, it is very important to understand the microstructure of perovskite and master the dynamic physical mechanism of photogenerated carriers. Transient absorption spectra (TAS) are a common means of studying ultrafast physical processes. In this paper, we study the ultrafast carrier dynamics of Cs_x_MA_1−x_PbI_3_ perovskite materials, mainly by using time-resolved transient absorption spectroscopy combined with X-ray diffraction (XRD) and UV–Visible absorption spectra, and attempt to elucidate their relaxation mechanism. The effects of different x-values (0%, 5%, 10%, 30%, 50%) on the crystal structure of mixed perovskite are discussed, and the dynamic physical behaviors of perovskite photogenerated carriers are analyzed. The transient absorption (TA) dynamics results revealed two channel mechanisms of carrier relaxation; the recombination lifetime of the 10% Cs^+^ doping perovskite was longer than that of pure MAPbI_3_. Three sets of time components were obtained by global fitting of the transient absorption spectra: hot carrier cooling, Auger recombination, and electron-hole recombination. The results showed that the photogenerated carriers of perovskite with 10% Cs^+^ doping have the longest relaxation time.

## 2. Experiment

### 2.1. Material Preparation

Cesium iodide (CsI), plumbum iodide (PbI_2_), and anhydrous N,N dimethylformamide (DMF) were purchased from Aladdin and used without further purification. Methyl ammonium iodide (CH_3_NH_3_I) was purchased from Shanghai MaterWin New Materials Co., Ltd. (Shanghai, China).

### 2.2. Preparation of Perovskite Materials

The synthesis of Cs^+^ doped perovskite films followed the slightly altered route of Choi, H. et al. [25]. Precursor solutions of Cs_x_MA_1−x_PbI_3_ were prepared by dissolving equimolar amounts of CsI: CH_3_NH_3_I and PbI_2_ in DMF at a concentration of 120 mg/mL. Solutions were exposed to ultrasonic treatment at 70 °C for 15 min, and then annealed for 10 min at 110 °C. Thus, the perovskite films were obtained. Preparation of samples: MAPbI_3_, Cs_0.05_MA_0.95_PbI_3_, Cs_0.1_MA_0.9_PbI_3_, Cs_0.3_MA_0.7_PbI_3_, Cs_0.5_MA_0.5_PbI_3_.

### 2.3. Experimental Characterization

**Figure 1:**
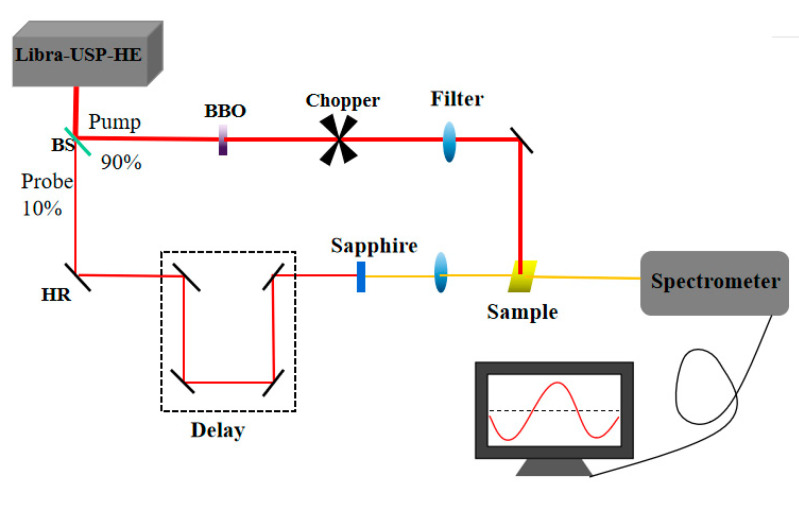
Experimental setup for transient absorption spectroscopy.

For XRD measurements, perovskite film diffractograms were collected using a PANalytical B.V., Empyrean at a voltage of 40 kV and current of 30 mA. The scanning angle range was 5–50°(2θ) with a rate of 0.3°/min. The UV–Vis absorption was measured using a UV-3101PC UV-VIS NIR scanning spectrophotometer. The laser system for TAS was a femtosecond titanium sapphire laser (Libra-USP-HE, Coherent Inc., Santa Clara, CA, USA) with a wavelength of 800 nm, a repetition rate of 1 kHz, a pulse width of 50 fs and an Ultrafast Systems (Helios) UV-visible detection system. The light was separated into pump (90%) and probe (10%) beams. The pump beam was directed through a Barium Boron Oxide crystal and a chopper (500 Hz) to produce the 400 nm pump beam, and the probe beam was passed through an optical delay rail and focused on a sapphire crystal to produce a white light continuum. In our transient absorption measurement, 400 nm with energy 3.1 eV was chosen as the pump excitation wavelength. Photoexcitation energy was attenuated to 0.88 μJ/cm^2^. All the transient absorption measurements were performed at room temperature. Figure 1 shows the setup of transient absorption.

## 3. Results and Discussion

### 3.1. Materials Crystal Characterization

**Figure 2:**
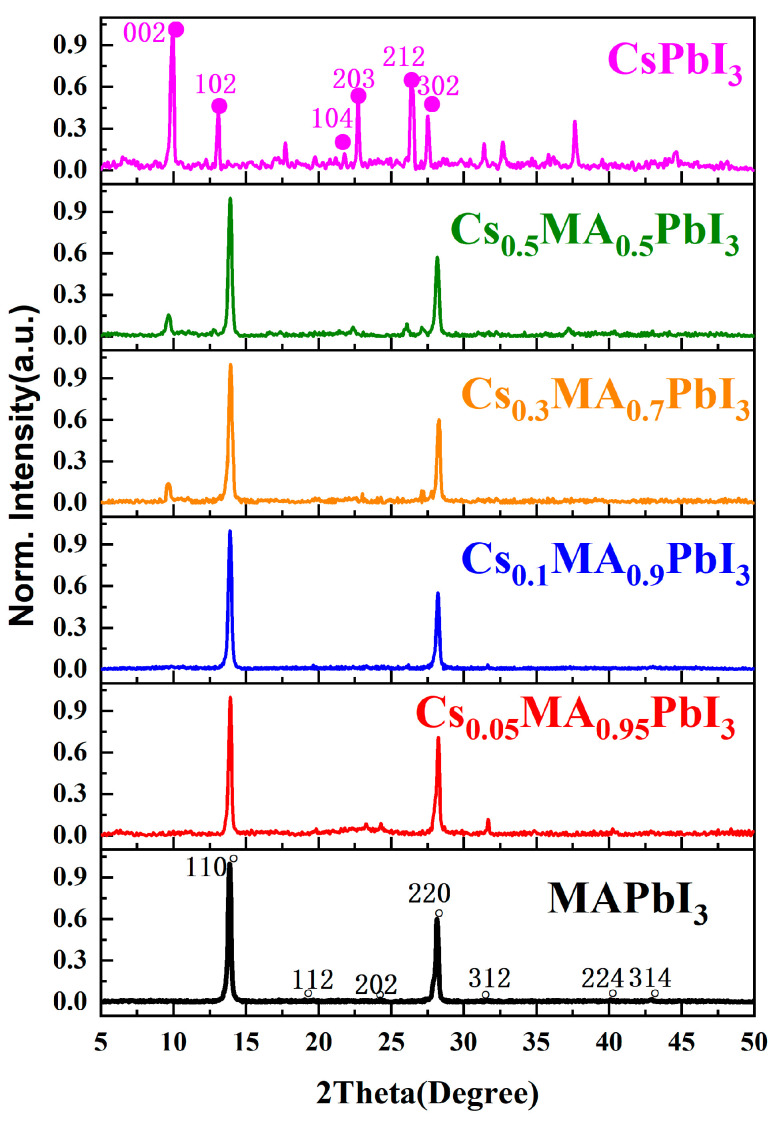
XRD spectra of Cs_x_MA_1−x_PbI_3_ (x = 0%, 5%, 10%, 30%, 50%, 100%) perovskite.

XRD was an effective method to study the microstructure of materials and could obtain some changes in the Cs_x_MA_1−x_PbI_3_ perovskite crystal phase at different x values. Figure 2 shows the X-ray diffraction scan map of Cs_x_MA_1−x_PbI_3_ (x = 0%, 5%, 10%, 30%, 50%, 100%). The pure MAPbI_3_ films exhibited diffraction peaks at 2θ values of 13.87°, 19.611°, 24.250°, 28.161°, 31.641°, 40.111°, and 42.871°, corresponding to the (110), (112), (202), (220), (312), (224) and (314) crystal planes of tetragonal perovskite, respectively [26,27,28]. For the pure CsPbI_3_ films, we observed strong diffraction peaks at 9.924°, 13.159°, 21.801°, 22.701°, 26.471°, and 27.530°, corresponding to the (002), (102), (104), (203), (212), and (302) crystal planes of the orthorhombic perovskite crystal structure [22,28,29,30]. Compared with the pure MAPbI_3_ perovskite, Cs^+^ replaced the A-site of the MAPbI_3_ perovskite, the position of the XRD diffraction peaks changed slightly with increasing x values in Cs_x_MA_1−x_PbI_3_. The lattice distortion and the lattice strain would cause changes in the position of diffraction peaks [22]. The peak intensity of CsPbI_3_ increased with the increase in the Cs^+^ ratio in Cs_x_MA_1-x_PbI_3_. In our experiments, XRD did not exhibit other peaks at x = 0–10%, proving that the tetragonal phase of MAPbI_3_ was maintained after Cs^+^ doping. When x = 30%, a small diffraction peak appeared at 9.92°, which corresponded to the orthorhombic phase CsPbI_3_ crystal structure; the diffraction patterns clearly showed both MAPbI_3_ and CsPbI_3_ crystal phases, indicating the presence of phase segregation. For this phenomenon, we believed that the both crystal phases existing in the Cs_x_MA_1−x_PbI_3_ films were caused by the excessive amount of Cs^+^ doping. It could also prove that Cs^+^ were successfully doped. Changes in x would cause a crystalline phase transition in the perovskite. The phase transition that was achieved depended on the [PbI_6_]^4−^ octahedra tilt and rotation in the lattice; meanwhile, the change in the [PbI_6_]^4−^ octahedral structure would affect the electronic orbital overlap state between Pb and I [31].

### 3.2. The UV-Visible Absorption Spectra

**Figure 3:**
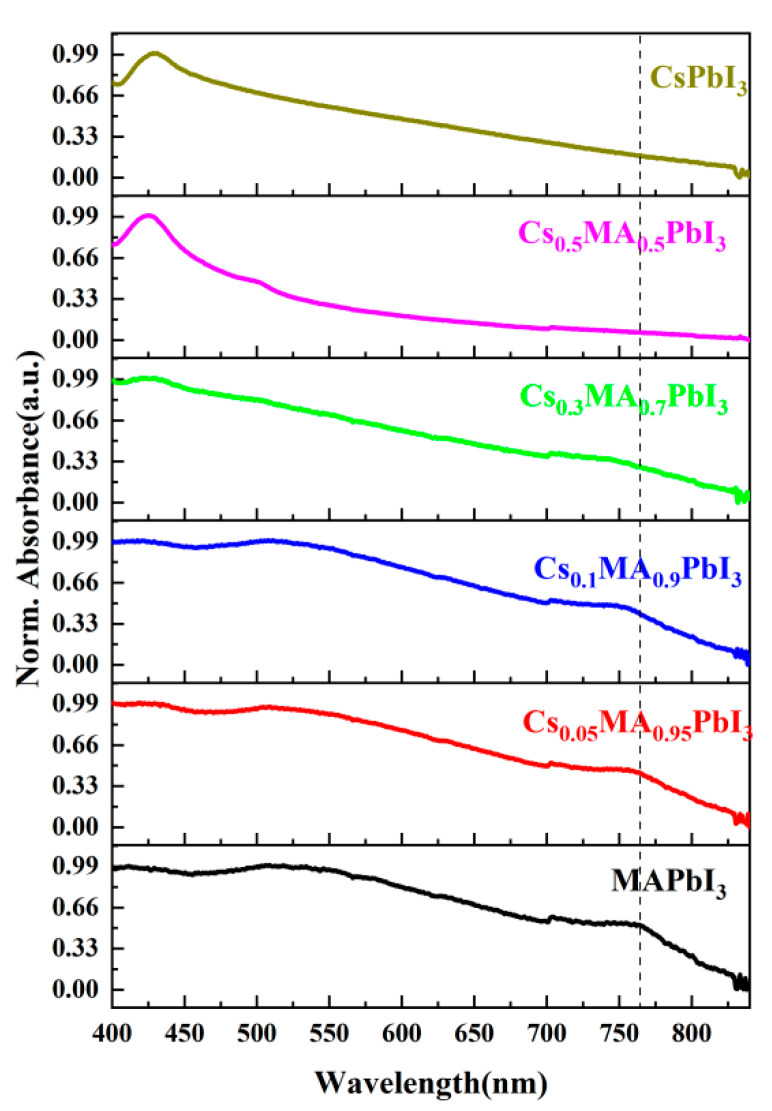
The UV-visible steady-state absorption spectrum of Cs_x_MA_1−x_PbI_3_ (x = 0%, 5%, 10%, 30%, 50%, 100%).

The steady-state absorption spectrum could observe the change in the band gap. Figure 3 shows the steady-state absorption spectrum of Cs_x_MA_1−x_PbI_3_; the pure CsPbI_3_ absorption peaks appeared at 430 nm, the band gap value was 2.8 ev. The pure MAPbI_3_ shows two broad absorption peaks at 500 nm and 760 nm, and the band gap value was 1.62 ev, which also matched the numerical value obtained by theoretical calculations of Taya, A. et al. and the band gap value obtained experimentally by Ayvazyan, G. Y. et al. [21,32]. Subsequent Cs^+^ doping had little effect on the band, so the Cs_x_MA_1−x_PbI_3_ absorption peak site did not change too much (band gap value was 1.62 ev for x = 5%; 1.63 ev for x = 10%). Increasing the amount of Cs^+^ could increase the absorption at 430 nm and decrease the absorption in the range of 500–760 nm. When x = 30%, the optical properties of the Cs_x_MA_1−x_PbI_3_ perovskite films began to reflect the absorption peaks properties in the wavelength range of 400–450 nm, suggesting that the optical properties began to reflect the peaks characteristics of the CsPbI_3_. This blue shift of the absorption peaks corresponded to an increase in the band gap of perovskite (band gap value was 1.67 ev for x = 30%; 1.71 ev for x = 50%). This result of the absorption peaks corresponded with that of the Choi, H. et al. [25]. MA^+^ had a strong electrostatic attraction to I^−^, which led to the distortion of the [PbI_6_]^4−^ octahedron in the tetragonal phase of MAPbI_3_ perovskite. We considered that doping a small amount of Cs^+^ could maintain the perovskite tetragonal phase when x = 0%, 5%, 10%. When x = 30%, 50%, 100%, the peak occurred at 400–450 nm, where we believed that because the CsPbI_3_ phase began to appear due to the increasing amount of Cs^+^ doping, the perovskite crystal phase changed from tetragonal phase to orthorhombic phase. After Cs^+^ doping, the H-bonding overall strength decreased somewhat, the [PbI_6_]^4−^ octahedron tilt increased, and there was enhanced coupling between the Pb-6s and the I-5p [21]. Therefore, the band gap change in the perovskite, owing to the [PbI_6_]^4−^ octahedral distortion, was caused by changes in the size of the A-site after Cs^+^ doping, rather than directly participating in the electronic structure of perovskite [33]. Our discussion was consistent with the results of X-ray diffraction (see Figure 2).

### 3.3. Carriers Dynamics for Transient Absorption Spectra

#### 3.3.1. Transient Absorption Spectra Characterization 

**Figure 4:**
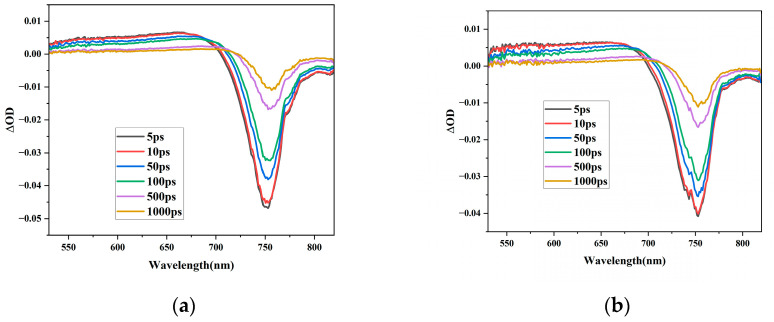
TAS at different delay times after excitation at 400 nm. (**a**) MAPbI_3_ perovskite and (**b**) Cs_0.1_MA_0.9_PbI_3_ perovskite.

The TAS technique is a powerful tool for studying excited-state relaxation dynamics. We performed TAS to investigate the excited state properties of Cs_x_MA_1−x_PbI_3_ perovskite films with different Cs^+^ ratios, where x varied from 0 to 1. Figure 4a,b shows the TAS of MAPbI_3_ and Cs_0.1_MA_0.9_PbI_3_ perovskite at different delay times, respectively. The TAS showed two main characteristic spectra: transient photo-induced absorption signals in the range of 520–700 nm; and a ground-state bleaching signal appearing around 750 nm, corresponding to the absorption bands showed by the steady-state absorption (Figure 3). After photoexcitation of perovskite, the photogenerated carriers occupied part of the conduction band (valence band); these filled states led to the generation of the ground-state bleaching signals. There was a consensus on the attribution of the ground-state bleaching signals at 750 nm [34], attributed to laser-induced band edge transition. The photo-induced absorption signals were accompanied by the ground-state bleaching signal. Cs_0.1_MA_0.9_PbI_3_ (Figure 4b) had a wider absorption width in the same delay time compared to MAPbI_3_. Furthermore, around 740 nm, a small spike was observed, which was due to the Cs^+^ doping changing the position and shape of the characteristic peaks at ground-state bleaching compared to MAPbI_3_.

#### 3.3.2. Single-Wavelength Evolution Dynamics

**Figure 5:**
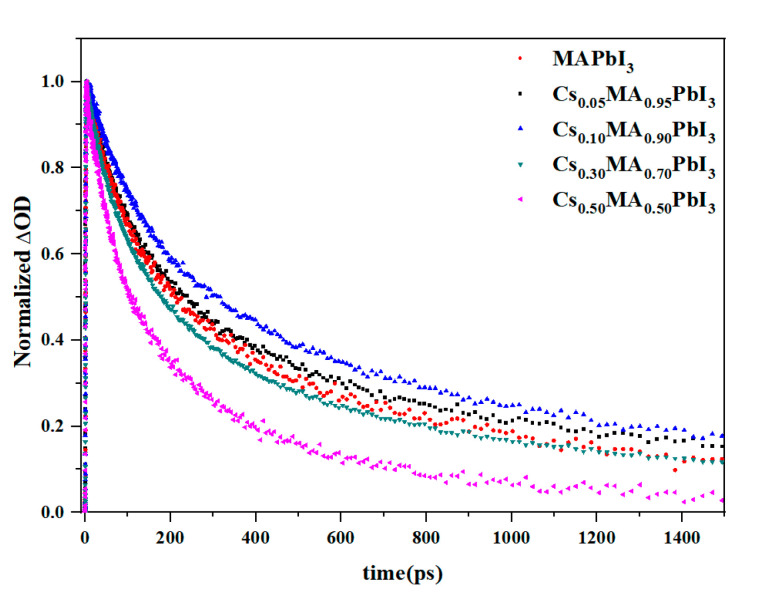
Transient decay response dynamics of Cs_x_MA_1−x_PbI_3_ at ground-state bleaching.

**Figure 6:**
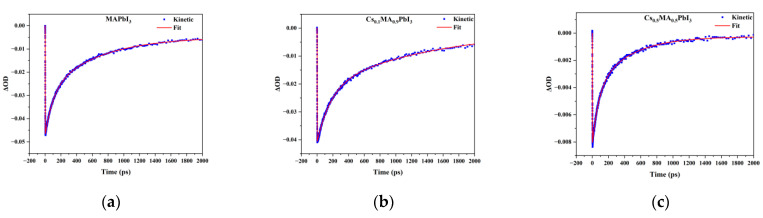
(**a**–**c**) are dynamic traces extracted of ground-state bleaching from the TAS of Cs_x_MA_1−x_PbI_3_ under different x value by kinetic fitting. (x = 0%, 10%, 50%).

The carriers density at the band gap was reasonably explained by the ΔOD intensity at the ground-state bleaching; it played a major role in the photophysical properties. To follow the carriers dynamics of perovskite with Cs^+^ doping, we monitored the transient decay dynamics of Cs_x_MA_1−x_PbI_3_ at the ground-state bleaching with different Cs^+^ doping amounts. Figure 5 shows the normalized transient decay response curve. Figure 6 shows the kinetic fit curve of Cs_x_MA_1−x_PbI_3_ at the ground-state bleaching at x = 0%, 10%, 50%. The double exponential function fitting could well match the decay curves of the Cs_x_MA_1−x_PbI_3_ perovskite, showing two recombination modes. Double exponential function:

$$S\left(t\right)=e^{{-(\frac{t-t_{0}}{t_{p}})}^{2}}*\left(A_{1}e^{-\frac{t-t_{0}}{t_{1}}}+A_{2}e^{-\frac{t-t_{0}}{t_{2}}}\right),\;t_{p}=\frac{IRF}{2\cdot \ln2}$$

where the IRF was the width of the instrument response function, t_0_ was the time zero, t_1_ and t_2_ were the time constants, and A_1_ and A_2_ represented the weighted contribution of each exponential component to the overall kinetics. For the kinetic fitting, the “infinite lifetime” component was used, and the resulting kinetic fitting constants were summarized in Table 1.

The two sets of time constants were obtained from the kinetic double exponential fitting of Cs^+^ doping the Cs_x_MA_1−x_PbI_3_ perovskite system. We attributed the faster decay process of carrier relaxation to Auger recombination. The slower decay carrier relaxation channel was attributed to electron-hole recombination. The carriers recombination strongly depended on the proportion of Cs^+^ doping. The Cs_x_MA_1−x_PbI_3_ perovskite with x = 10% had the largest carrier lifetime, which meant the best performance. Compared with lower amount of Cs^+^ doping (x = 5%, 10%) and pure MAPbI_3_ perovskite, the recombination lifetime of the Cs_x_MA_1−x_PbI_3_ perovskite with a high amount of Cs^+^ doping (x = 30%, 50%) was faster. The introduction of high concentrations of Cs^+^ accelerated the process of carrier recombination. However, the dynamics of the carriers extracted by the local fitting method at the ground-state bleaching might be relatively inaccurate; the global analysis tracked the evolution of the full spectrum.

#### 3.3.3. Full Spectrum Evolutionary Dynamics

**Figure 7:**
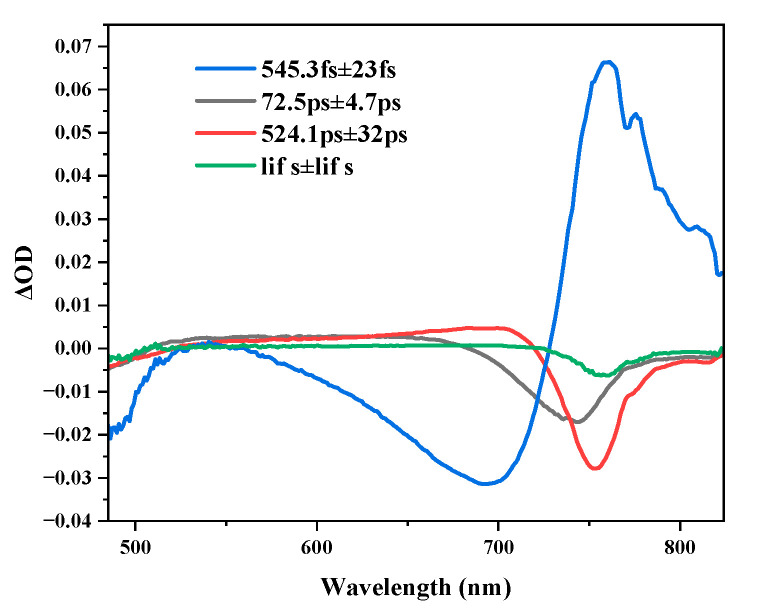
MAPbI_3_ decay correlation spectra obtained by global fitting.

The dynamic processes of Cs_x_MA_1−x_PbI_3_ was mainly affected by carrier cooling and recombination processes. Cs_x_MA_1−x_PbI_3_ perovskite carriers absorbed the pump energy and then jumped to higher energy states. Carrier instability in an excited state would go back to the ground state through a series of relaxation processes. TAS contained very complex components. In order to strip these independent carrier’s physical processes and extract the corresponding physical information, taking pure MAPbI_3_ as an example, we used the singular value decomposition (SVD) to isolate the major components of perovskite [35] where independent observations of the temporal evolution of the complex TA spectra became possible, and performed a global fitting operation to obtain three sets of time constants: 550.3 fs, 71.9 ps, 521.9 ps. Restricted by the experimental instrument, we found that the carriers did not attenuate completely in the measurement time range, we added an infinite exponent in the global fitting process. Figure 7 shows the spectral information obtained from the global fitting. In the early stage of photoexcitation, carrier energy was redistributed (thermalized) on time scales on the order of femtosecond, satisfying the Fermi Dirac’s statistical law [36,37]. Temperature mismatch between hot carriers and lattices, and hot carrier cooling was achieved by interaction with lattices. Hot carrier cooling relaxed to a quasi-thermal equilibrium state, mainly through scattering longitudinal optical (LO) phonons; the process was usually very fast, as shown in the subpicosecond time scales [38,39]. The hot optical phonons generated by the carrier relaxation process would further collide and scatter with acoustic phonons, and thus re-establish the thermal equilibrium state between the phonons [38]. During the cooling process of the carriers, the carriers collide with the lattices and exchange energy; the lattice’s vibration in the crystal was described by a quantized phonon. Therefore, the carrier cooling process could also be understood as the process of a carriers–phonons interaction. Thus, the 550.3 fs was assigned to the process of carrier cooling. Auger recombination and electron-hole recombination contributed to carrier recombination dynamics. Manser et al. suggested that carrier recombination in MAPbI_3_ was mainly through electron-hole recombination [40]. Shen et al. pointed out that the spin-orbit coupling of the Pb-based perovskite would induce spin splitting in the degenerate energy level at the conduction band edge; MAPbI_3_ spin-splitting energy only resonated with the band gap energy, which was responsible for the existence of the Auger recombination of MAPbI_3_ [41]. The relevant processes appeared on different time scales, with the Auger recombination process occurring on tens of picosecond scales [42]. The Auger recombination process was also an important source of carriers thermalization. In the Auger recombination process, the excess energy would be transferred to the third carrier, making the third carrier transition to a higher energy level, the resulting thermal effect would also delay the carriers cooling process, so the recombination process was also called the Auger heating process. We found that the spectral information obtained from the global fitting (Figure 7), where the 72.5 ps time component signal overlapped to varying degrees with the 545.3 fs cooling mechanism and the 524.1 ps time component signal, was a good indication of the 72.5 ps Auger heating phenomenon. The Auger heating phenomenon was involved in the cooling and recombination mechanisms [43]. Therefore, the 72.5 ps was assigned as the Auger recombination process, and the 524.1 ps was assigned as the result of electron-hole recombination. The model proposed by Jia et al. could help us to better understand the carriers relaxation process [44].

**Figure 8:**
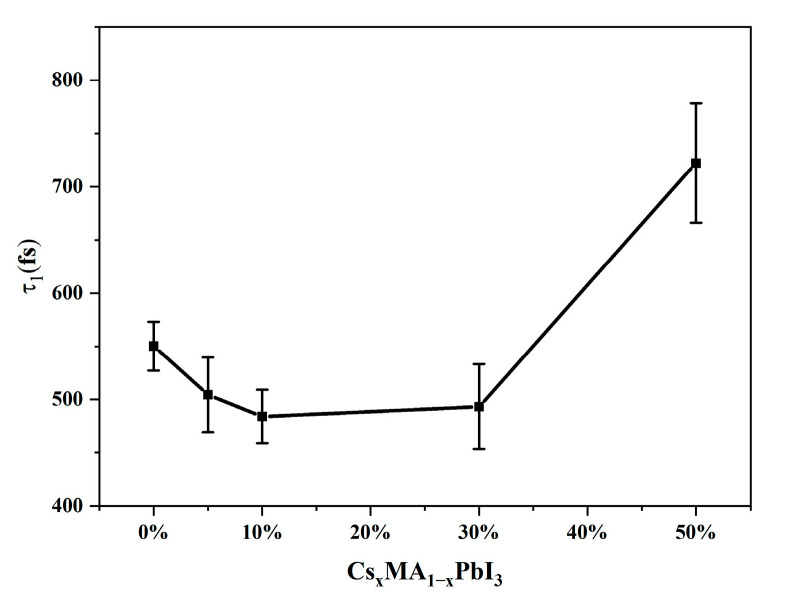
The hot carriers cooling time constant with different x values obtained from global fitting to the TAS of Cs_x_MA_1−x_PbI_3_.

In order to better develop the application of perovskite in solar cells, it was very important to understand the hot carrier cooling process. In order to clearly understand the influence of Cs^+^ doping on the cooling process of Cs_x_MA_1−x_PbI_3_ carriers, Figure 8 showed the trend of the hot carrier cooling time constant with different x values. The presence of electrostatic attraction between MA^+^ and I^−^ caused the distortion of [PbI_6_]^4−^ octahedral framework in the tetragonal phase MAPbI_3_. Cs^+^, as a cation with isotropic charges distribution, formed stronger and more uniform electrostatic attraction with [PbI_6_]^4−^ octahedra to maintain the perovskite structure. A small amount of Cs^+^ doping could maintain the perovskite phase. In x ≤ 10% interval, for the process of cooling time constant reduction, we could understand the phenomenon; a smaller Cs^+^ radius caused stronger interactions between the A-site cation and the [PbI_6_]^4−^ octahedron in the perovskite, leading to a larger LO phonons energy [45]. The larger LO phonons energy facilitated energy transfer from hot carriers to LO phonons through the hot carrier–LO phonons scattering process, and bound organic cation MA^+^ was not conducive to acoustic phonon up-conversion efficiency [46], resulting in a decrease in the cooling time constant. At x ≥ 10%, the CsPbI_3_ crystal‘s phase features were displayed in the system. A small diffraction peak at x = 0.3 appeared at 9.28° for the XRD, measured by Imran, M. et al. [22], which also indicated that phase segregation occurred at 10% Cs^+^ doping amount due to the severe [PbI_6_]^4−^ distortion of octahedra, which would favor the generation of large polaritons. The formation of polaritons in perovskite was thought to effectively protect thermal carriers from scattering by phonons [47], leading to an increase in cooling time.

**Figure 9:**
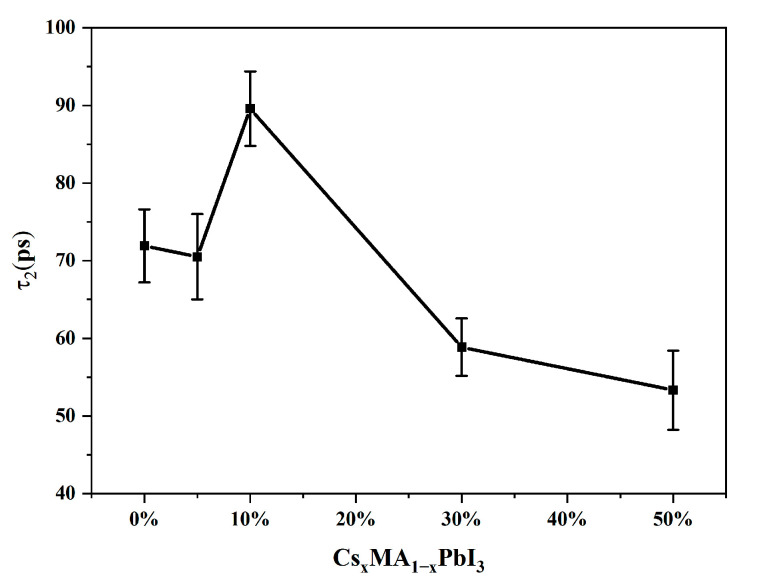
The Auger recombination time constant with different x values obtained from global fitting to the TAS of Cs_x_MA_1−x_PbI_3_.

τ_2_ was the Auger recombination time constant. With the increase in x, the Auger recombination time constant appeared to increase and then decrease (Figure 9); the turning point occurred at x = 10%. The XRD of pure MAPbI_3_ indicated a tetragonal phase, where structural symmetry was excellent, and no new diffraction peaks appeared at x < 10%, which also indicated that a small amount of Cs^+^ doping did not change the crystal structure [48]. We speculated that it would affect the crystal symmetry, and the spin-orbit coupling in MAPbI_3_ would lead to a splitting of the energy band, which was associated with MAPbI_3_ structural instability, leading to a stronger Auger recombination [41]. A small amount of Cs^+^ doping was able to maintain the crystal structure of MAPbI_3_, thus inhibiting the lattice distortion, resulting in the Auger recombination time constant at 10% Cs^+^ doping greater than MAPbI_3_. All of this led to slow the decay rate and increase the recombination time. At x > 10%, we mentioned that the rise of the perovskite band gap was caused by the [PbI_6_]^4−^ octahedral structural distortion, which had a positive effect on the Auger recombination, and could be traced in the work of Phuong et al. [49]. The enhanced band splitting of the [PbI_6_]^4−^ octahedral distortion also directly affected the state distribution involved in the Auger process [41]. This was also a good reason why the Auger recombination time constant at high Cs^+^ doping was lower than that of pure MAPbI_3_. Therefore, the Auger recombination time constant decreased in this range.

**Figure 10:**
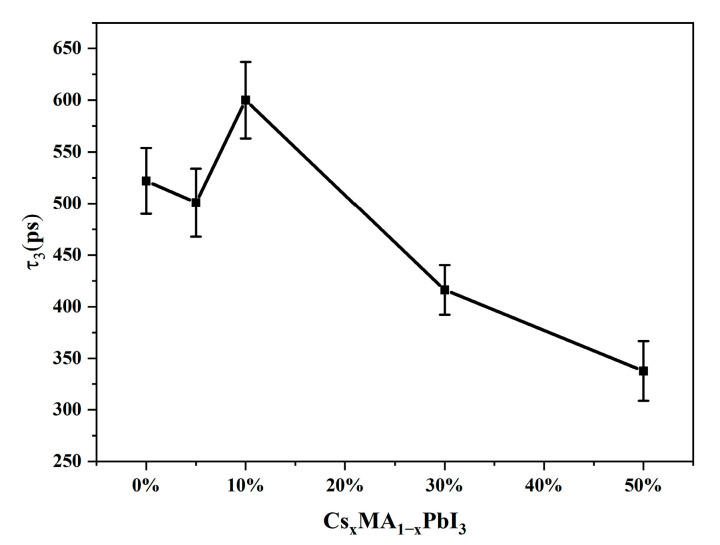
The electron-hole recombination time constant with different x values obtained from global fitting to the TAS of Cs_x_MA_1−x_PbI_3_.

For the τ_3_ electron-hole recombination process (Figure 10), it could be concluded that the time constant of the perovskite system also appeared to increase and then decrease as x increased; the turning point occurred at x = 10%. It had been proposed that a small amount of Cs^+^ doping could passivate the defective state. The mismatch between the doped atoms and the lattice caused an increase in defective states when the Cs^+^ doping amount was too much [41]. The defective state was able to capture photogenerated carriers and stimulated recombination at the defective site, thus accelerating the carriers recombination process [50]. At x < 10%, we considered that the defective state was passivated in perovskite, which prevented the carriers from being trapped by the process, the electron-hole recombination time constant increased. When Cs^+^ doping amount reached a certain degree, the crystal structure changed. The CsPbI_3_ phase began to appear in the system, which introduced more grain boundaries and defects [51,52,53], which leaded to a massive of carriers trapping by defective states, and there were unavoidable trap sites at these grain boundaries introduced by increased Cs^+^, which could also serve as carrier recombination centers, thus electron-hole recombination time decreased. Different experiments consistently showed that Cs^+^ doping could improve the uniformity of the Cs_x_MA_1−x_PbI_3_ perovskite and maintain the crystal phase of perovskite. When the Cs^+^ doping amount was too large, it began to destroy the growth of internal grains [22,23,28,54]. Better crystal quality was also responsible for the increased lifetime. Surprisingly, for the x = 10% Cs^+^ doping, the Auger recombination lifetime and electron-hole recombination lifetime of the Cs_x_MA_1−x_PbI_3_ perovskite exhibited a longer carrier lifetime than that of pure MAPbI_3_ perovskite, which had potential applications in improving the photovoltaic conversion efficiency of solar cells. Our results showed that Cs_x_MA_1−x_PbI_3_ perovskite materials had the longest relaxation time and performed the best in solar cells at x = 10%.

## 4. Conclusions

Applying TAS to elucidate the free carrier dynamics of pure the MAPbI_3_ perovskite and mixed cation perovskites uncovered the underlying reasons for their excellent performance and high stability. The dynamics at ground-state bleaching revealed two channel mechanisms of carriers relaxation: Auger recombination and electron-hole recombination, with a time constants of 80–179 ps and 425–1502 ps, respectively. Three sets of time constants were obtained by SVD and global fitting: hot carrier cooling, Auger recombination and electron-hole recombination. A small amount of Cs^+^ doping could maintain the perovskite structural crystal phase. When Cs^+^ doping was performed in excess, it would change the MAPbI_3_ crystal phase, which originated in [PbI_6_]^4−^ octahedral structural distortion caused by Cs^+^ doping, then the photogenerated carrier’s lifetimes were affected. Our results showed that Cs_x_MA_1−x_PbI_3_ perovskite had superior photophysical properties at 10% Cs^+^ doping, and were candidates for highly efficient solar cell applications. Our experiments provided an experimental basis for the application of MAPbI_3_, and we expect to further explore the application of perovskite materials.

## Figures and Tables

**Table 1:** Single-wavelength double exponential decay kinetic fit of Cs^+^ doping Cs_x_MA_1-x_PbI_3_ perovskite.

	Wavelength (nm)	τ_1_ (ps)	τ_2_ (ps)
MAPbI_3_	750	104.1 ± 14.7 (43%)	652.8 ± 131 (57%)
Cs_0.05_MA_0.95_PbI_3_	750	110.4 ± 18.4 (45%)	679.4 ± 174 (55%)
Cs_0.1_MA_0.9_PbI_3_	750	178.5 ± 15 (48%)	1502 ± 130 (52%)
Cs_0.3_MA_0.7_PbI_3_	736	84.11 ± 12 (44%)	512.7 ± 82 (56%)
Cs_0.5_MA_0.5_PbI_3_	720	80.1 ± 15 (49%)	424.5 ± 101 (51%)

## Data Availability

Data are contained within the article.
